# Diversity of Bacterial Biota in *Capnodis tenebrionis* (Coleoptera: Buprestidae) Larvae

**DOI:** 10.3390/pathogens8010004

**Published:** 2019-01-06

**Authors:** Hana Barak, Pradeep Kumar, Arieh Zaritsky, Zvi Mendel, Dana Ment, Ariel Kushmaro, Eitan Ben-Dov

**Affiliations:** 1 Avram and Stella Goldstein-Goren Department of Biotechnology Engineering, Ben-Gurion University of the Negev, P.O. Box 653, Beer-Sheva 8410501, Israel; hanare@post.bgu.ac.il (H.B.); pkbiotech@gmail.com (P.K.); arielkus@bgu.ac.il (A.K.); 2 Faculty of Natural Sciences, Ben-Gurion University of the Negev, P.O. Box 653, Beer-Sheva 8410501, Israel; ariehzar@gmail.com; 3 Department of Entomology, Agricultural Research Organization, The Volcani Center, Rishon LeZion 7505101, Israel; zmendel@volcani.agri.gov.il (Z.M.); danam@volcani.agri.gov.il (D.M.); 4 National Institute for Biotechnology in the Negev, Ben-Gurion University of the Negev, Beer-Sheva 8410501, Israel; 5 Department of Life Sciences, Achva Academic College, M.P. Shikmim Arugot 7980400, Israel

**Keywords:** bacterial biota, Buprestidae, *Capnodis*, stonefruit

## Abstract

The bacterial biota in larvae of *Capnodis tenebrionis*, a serious pest of cultivated stone-fruit trees in the West Palearctic, was revealed for the first time using the MiSeq platform. The core bacterial community remained the same in neonates whether upon hatching or grown on peach plants or an artificial diet, suggesting that *C. tenebrionis* larvae acquire much of their bacterial biome from the parent adult. Reads affiliated with class levels *Gammaproteobacteria* and *Alphaproteobacteria* (phylum *Proteobacteria* ca. 86%), and *Actinobacteria* (ca. 14%) were highly abundant. Most diverse reads belong to the families *Xanthomonadaceae* (50%), *Methylobacteriaceae* (20%), *Hyphomicrobiaceae* (9%), *Micrococcaceae* (7%) and *Geodermatophilaceae* (4.5%). About two-thirds of the reads are affiliated with the genera *Lysobacter*, *Microvirga*, *Methylobacterium*, and *Arthrobacter*, which encompass species displaying cellulolytic and lipolytic activities. This study provides a foundation for future studies to elucidate the roles of bacterial biota in *C. tenebrionis*.

## 1. Introduction

Insect pests may pose significant challenges to environmental quality and human welfare [1], but contribution of their symbiotic microorganisms for establishment and cause additional environmental and economic injury is still vague [2]. Multispecies microbial communities harbored in insect guts are involved in nutrition, digestion, and defense activities. Little is known about the diversity, physiology, and ecology of microorganisms associated with bark and wood-boring beetles [3,4]. Insect-symbiotic bacteria supplement essential nutrients, degrade complex dietary polymers and plant toxins [5,6,7], and thus contribute to overcoming plant defenses and higher host fitness [8,9]. Flatheaded borers *Capnodis* spp. (Coleoptera: Buprestidae) inflict serious harm to fruit and ornamental trees around the Mediterranean, in Southern Europe and in Western Asia [10]. Control regimes of *Capnodis* spp. populations rely on intensive applications of synthetic insecticides, whereas environmentally friendly means are partially absent. *Capnodis tenebrionis* is the most notorious pest among the congeners. Revealing the bacterial biota in larvae may be an avenue to the development of new, safe tools to cope with this pest. Information about microorganisms harbored by *C. tenebrionis* larvae does not exist; studying their core microbiota can improve the ability to develop more effective management approaches for this pest [11].

## 2. Results and Discussion

**Figure 1:**
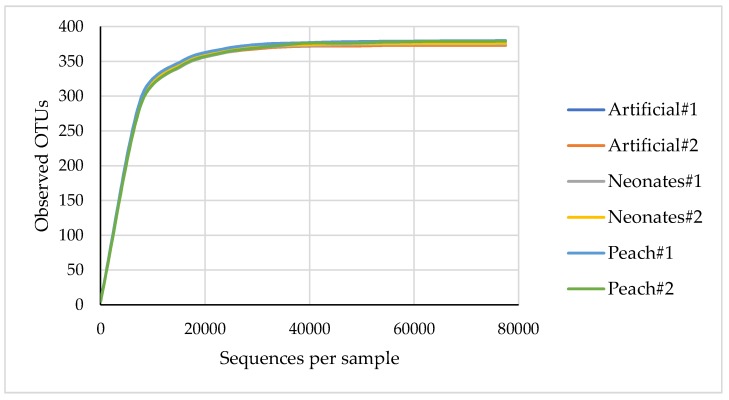
Rarefaction curves represent observed OTUs from *C. tenebrionis* under different feeding regimes.

The number of reads per sample that passed a set of sequence filters range from 106,249 to 144,439, with lengths of about 495 bp. The rarefaction analysis allowed for a comparison of species richness (number of OTUs) between different larvae and a determination of adequacy of sequencing output for each feeding regime. All rarefaction curves show the same slope and reach the plateau with 30,000 reads per sample (Figure 1).

**Figure 2:**
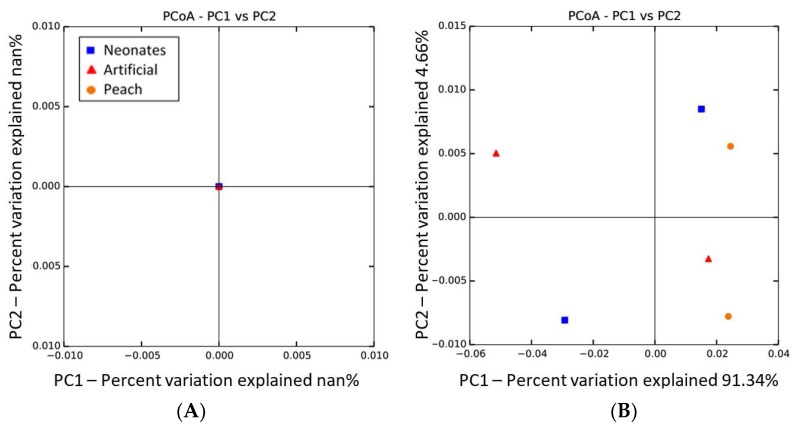
Bacterial community structures of larvae that grew on peach plants or an artificial diet, and in neonates. (**A**) UniFrac-unweighted principal coordinate analysis and (**B**) UniFrac-weighted principal coordinate analysis.

The unweighted and weighted UniFrac phylogenetic distance metric plots, obtained using a principal coordinate analysis (PCoA), allowed for an assessment of microbial community differences between larvae of *C*. *tenebrionis*, neonates, and those grown on either peach plants or an artificial diet (Figure 2). The unweighted UniFrac distances (Figure 2A) show that compositions of these samples were identical, but the weighted distances (Figure 2B) display a variance in the communities’ composition that is unexplained by diet type. Thus, diet type seems not to affect the bacterial biota composition of *C. tenebrionis* larvae. In other species, larval diet does affect the microbiota composition [6,12]. The pine weevil beetle *Hylobius abietis* is, however, also resilient to changes in diet [9]: their microbiome is the same whether they were fed on an artificial diet or on Norway spruce twigs.

**Figure 3:**
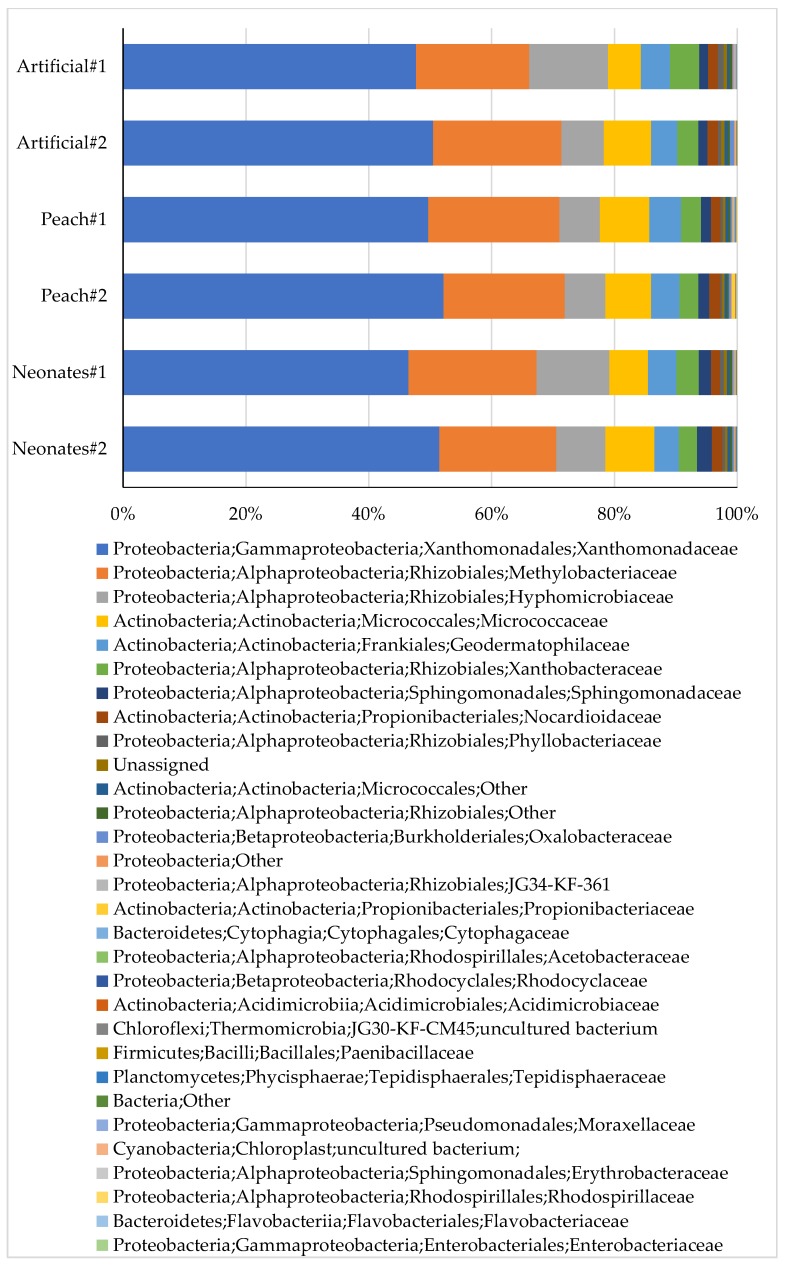
Taxonomy assignment at the family level of *C. tenebrionis* on three different feeding regimes.

Most abundant reads in all *C. tenebrionis* larvae were affiliated with phyla of *Proteobacteria* (84–87%) and *Actinobacteria* (12–15%) (Figure 3; Table 1). At the class level, they were assigned to *Gammaproteobacteria* (46–52%), *Alphaproteobacteria* (32–39%), and *Actinobacteria* (12–15%) (Figure 3; Table 2).

The most abundant (ca. 50%) reads were affiliated with the *Xanthomonadaceae* family within the *Gammaproteobacteria* class (Figure 3), where 45% of them were not identified at the genus level using the Silva database as a reference. Blastn analysis (https://blast.ncbi.nlm.nih.gov/Blast.cgi), however, revealed that these reads are affiliated with genus *Lysobacter*, members of which are Gram negatives widely distributed in soil, plant, and freshwater habitats. Reads affiliated with *Lysobacter* were one of the most prevalent groups in the gut flora of herbivorous cephalotine ants (*Cephalotes varians*, *C. rohweri*, and *C. atratus*) [13]. Cellulase and glucanase activities [14] were identified in some *Lysobacter* species: IB-9374 [15], *L. capsici* AZ78 [16], and *L. enzymogenes* strain N4-7 [17]. Moreover, *L. enzymogenes* controls phytopathogenic nematodes [18] and fungi such as *Bipolaris sorokiniana* [19], *Uromyces appendiculatus* [20], *Fusarium graminearum* [21], and *Rhizoctonia solani* [22]. *Lysobacter gummosus* that lives on redback salamanders’ skin produces 2,4-diacetylphloroglucinol, which inhibits the growth of certain pathogenic fungi [23].

The second most abundant (ca. 20%) reads are affiliated with the *Methylobacteriaceae* family (order *Rhizobiales*, class *Alphaproteobacteria*) (Figure 3). The blastn revealed that 91% (ca. 65%, similarity cut-off 96%; 26%, similarity cut-off 97%) of the all *Methylobacteriaceae* are affiliated with genus *Microvirga*, 7.5% are classified as genus *Methylobacterium*, and the rest (about 1.5% reads) were identified as uncultured *Methylobacteriaceae*.

The *Microvirga* (formerly *Balneimonas*) and *Methylobacterium* species are ubiquitous in nature, mainly soil and water, but also plants’ phylloplane [24,25,26]. Bacteria of the *Microvirga* and *Methylobacterium* genera have recently been found essential for efficient digestion of lignocellulose in the gut of the wood-feeding termite *Reticulitermes chinensis* (Isoptera: Rhinotermitidae) [27], that digest glucosyl and xylosyl residues from lignocellulose [28]. *Methylobacterium* spp. perform lipolytic activity in weevils (Coleoptera: Curculionidae) and may play a role in nutritional processes [29]. The abundance (ca. 20%) of *Microvirga* and *Methylobacterium* in *C. tenebrionis* may therefore be involved in cellulolytic and lipolytic activities.

The third most abundant (ca. 9%) reads are affiliated with the *Hyphomicrobiaceae* family within the *Alphaproteobacteria* class (Figure 3). Analysis by blastn classified them to the genus *Devosia* (similarity cut-off 96%). Cellulolytic bacteria belonging to this genus were isolated from the larval gut of root-feeding *Holotrichia parallela* (Coleoptera: Scarabaeidae) [30] and of silkworm [31]. It is reasonable that the flat-headed root borer *C. tenebrionis* harbors these cellulolytic bacteria.

The fourth most abundant (7%) reads are affiliated with the *Micrococcaceae* family (class *Actinobacteria*) (Figure 3). Analysis by blastn affiliated most of them with the Gram-positive coryneform genus *Arthrobacter* (similarity cut-off 97%), members of which are commonly found in soil. *Arthrobacter gandensis* and *A. gandavensis* were isolated from the wireworm *Agriotes lineatus* (L.) (Coleoptera: Elateridae), a serious pest of various vegetables and fruits throughout the world [32]. *Arthrobacter pityocampae* was isolated from the pine processionary moth *Thaumetopoea pityocampa* (Lepidoptera: Thaumetopoidae), one of the most harmful pests of pine species in Mediterranean countries [33]. *Arthrobacter* ssp. are prevalent in the gut microbiota of the cave beetles *Neobathyscia pasai* and *N*. *mancinii* (Coleoptera: Leiodidae) [34]. Wood-digesting *Arthrobacter* sp., which had been isolated from the hind-gut of the termite *Reticulitermes hesperus* [35], was also found in the microbiome of *C. tenebrionis*.

The fifth most abundant (4.5%) reads are affiliated with the *Geodermatophilaceae* family (order *Actinomycetales*) (Figure 3). Analysis by blastn affiliated most of them with the genus *Blastococcus* (similarity cut-off 97%). Members of the family *Geodermatophilaceae* contain bacteria isolated mainly from soils, seawater, and stone surfaces [36]. Little is known about *Blastococcus*, but reads belonging to this genus have been retrieved from globally important pest, the chilli thrips *Scirtothrips dorsalis* (Thysanoptera: Thripidae) [37] and strains affiliated with *Geodermatophilus*, were found associated with *Paratrechina longicornis* (Hymenoptera: Formicidae) [38].

Apart from habitat- and diet-specific microbes, an insect’s gut harbors a core microbiome, members of which have likely co-evolved with the host and fulfill important functions, such as cellulose degradation [39], breakdown of ingested toxins, or overcome chemicals used for insect control [6,40,41]. Indigenous bacteria are often specialized gut symbionts and are transmitted vertically from the eggs, through coprophagy or social interactions. Gut communities of social insects are usually more distinctive and consistent than those of non-social invertebrates [6]. The findings described here reveal a high similarity of microbial communities retrieved from *C. tenebrionis* neonates (hatching larvae before any act of feeding) or reared on either peach plants or an artificial diet. These findings imply that the larvae acquire much of their bacterial biome from the parent adult.

Gut symbionts may have the potential to protect their host from insecticides such as fenitrothion [40]. In gypsy moth larvae, on the other hand, elimination of the indigenous midgut microbial community abolished insecticidal activity of Cry’s, and re-introduction of a specific member of this community restored *Bacillus thuringiensis*-mediated killing [42]. Bacterial symbionts may be utilized to manage insect pests [6] in different ways: insecticidal potential of entomopathogenic gut bacteria may serve for pest management; genetically modified bacteria can be used as vehicles to specifically express foreign traits that interfere fitness of the pest; gut symbiont that naturally inhibit parasite colonization could be disseminated in insect populations, for example, to prevent spread of human disease via insect vectors or influence vector competence by modulating immune responses. Characterizing the core microbiota of *C. tenebrionis* larvae is essential for understanding their physiology and ecology, and thus could be helpful in developing the next generation of pest control strategies.

## 3. Materials and Methods

Adults of *C. tenebrionis* were collected in nectarine orchard next to Yesod HaMa’ala (Hulla Valley, 33°05′26″ N, 35°58′90″ E, and newly-hatched larvae of the first lab generation were examined. Larvae were randomly sampled from hatching neonates, and from individuals lab-reared on peach plants and on an artificial diet over four weeks [43]. The larvae were surface-sterilized with 75% ethanol for 90 s, rinsed twice with sterilized-deionized water, and stored at −80 °C.

Total bacterial DNA was extracted from the larvae using the PowerSoil^®^ DNA Isolation Kit (MO BIO laboratories, San Diego, CA, USA) following the manufacturer’s protocol, except that each sample was placed into liquid nitrogen and crushed with a pestle before cell lysis. Total genomic DNA extraction of 6 samples, each composed of 3 *C. tenebrionis* larvae, was performed by the DNA Services Facility at the Research Resources Center, at the University of Illinois at Chicago, for 16S rRNA gene sequencing using the Illumina MiSeq platform. Conserved regions V1–V3 of the 16S rDNA were amplified using PCR with the pair of primers CS1_27F/CS2_534R. A total of 1,056,756 reads were obtained.

Raw reads were merged using the software package PEAR (v0.9.10) [44]. Low quality sequences and chimeras were removed by the software package Mothur (v1.36.1) [45]. The quality-controlled sequences were processed with the Quantitative Insights into Microbial Ecology (QIIME v1.9.1) package [46]. Briefly, sequence data were clustered into operational taxonomic units (OTU) at 97% similarity using UCLUST. Representative sequences from each OTU were extracted and aligned using PyNAST with a percent identity threshold of 60% to the Silva 16S rRNA bacterial database [47]. Representative sequences were classified for taxonomy assignment by UCLUST and Silva database. Unassigned OTUs at the genus level were analyzed using standard nucleotide BLAST analysis (https://blast.ncbi.nlm.nih.gov/Blast.cgi). A Biological Observation Matrix (BIOM) was generated at different taxonomic levels. OTU with a total observation count lower than 50 reads were discarded. Qiime was also used to generate an alpha rarefaction plot as well as principal coordinate analysis (PCoA) plots based on weighted and unweighted UniFrac metrics (beta diversity).

## Figures and Tables

**Table 1:** Taxonomy assignment at the phylum level of *C. tenebrionis* on different feeding regimes.

Phylum	Artificial#1	Artificial#2	Neonates#1	Neonates#2	Peach#1	Peach#2
*Proteobacteria*	87.22%	85.12%	86.60%	85.21%	83.86%	84.69%
*Actinobacteria*	12.09%	14.25%	12.83%	14.23%	15.38%	14.85%
Unassigned	0.54%	0.56%	0.49%	0.37%	0.42%	0.35%
Others ^1^	0.15%	0.07%	0.08%	0.18%	0.34%	0.11%

^1^ Others = Bacteroidetes, Chloroflexi, Firmicutes, Planctomycetes, Cyanobacteria.

**Table 2:** Taxonomy assignment at the class level of *C. tenebrionis* on different feeding regimes.

Class	Artificial#1	Artificial#2	Neonates#1	Neonates#2	Peach#1	Peach#2
*Gammaproteobacteria*	47.69%	50.47%	46.49%	51.49%	49.67%	52.31%
*Alphaproteobacteria*	39.13%	33.80%	39.65%	33.28%	33.79%	31.88%
*Actinobacteria*	12.07%	14.23%	12.79%	14.21%	15.35%	14.83%
Unassigned	0.54%	0.56%	0.49%	0.37%	0.42%	0.35%
Others ^1^	0.58%	0.94%	0.57%	0.64%	0.77%	0.63%

^1^ Others = Betaproteobacteria, Cytophagia, Acidimicrobiia, Thermomicrobia, Bacilli, Phycisphaerae, Chloroplast, Flavobacteriia.
